# Monitoring Rates for Metabolic Syndrome in Adults Treated With Atypical Antipsychotics: A Population-Based Study in British Columbia: Fréquence de la surveillance du syndrome métabolique chez les adultes traités par des antipsychotiques atypiques : étude fondée sur la population menée en Colombie-Britannique

**DOI:** 10.1177/07067437261420884

**Published:** 2026-02-13

**Authors:** Ridhwana Kaoser, M. Ruth Lavergne, Sandra Peterson, Agnes Grudniewicz, Helen Thai, Lucie Langford, Rita K. McCracken, Sara English, David Rudoler

**Affiliations:** 1 Faculty of Health Sciences, 1763 Simon Fraser University, Vancouver, British Columbia, Canada; 2 Department of Family Medicine, Primary Care Research Unit, 3688 Dalhousie University, Halifax, Nova Scotia, Canada; 3 Centre for Health Services and Policy Research, 8166 The University of British Columbia, Vancouver, British Columbia, Canada; 4 Telfer School of Management, 6363 University of Ottawa, Ottawa, Ontario, Canada; 5 Department of Psychology, 5620 McGill University, Montreal, Quebec, Canada; 6 Centre for Studies in Primary Care (CSPC), Department of Family Medicine, 4257 Queen's University, Kingston, Ontario, Canada; 7 Faculty of Medicine, Department of Family Practice, 12358 UBC, Vancouver, British Columbia, Canada; 8 Faculty of Medicine, 12361 Dalhousie University, Halifax, Nova Scotia, Canada; 9 85458 Ontario Shores Centre for Mental Health Sciences, Whitby, Ontario, Canada; 10 Faculty of Health Sciences, Ontario Tech University, Oshawa, Ontario, Canada

**Keywords:** preventive medicine, metabolic syndrome, antipsychotic agents, adverse effects, drug monitoring

## Abstract

**Purpose:**

We examine the rate of recommended screening and monitoring practices for detecting metabolic changes following treatment with atypical antipsychotics, comparing data from the pre-pandemic and post-pandemic periods. We also compared the characteristics of adults who undergo screening tests with those who did not to identify underserved populations.

**Method:**

We use population-based administrative data from British Columbia, Canada, between 2018 and 2023 to describe the characteristics of adults who received or did not receive the laboratory test within recommended timeframes. To identify baseline screening tests, the cohort was stratified into antipsychotic-naïve and non-naïve groups. We describe the number and percentage of each lab test completed by patients, and the specialty of the ordering provider across different periods.

**Results:**

Over one-third of the population did not receive any recommended screening tests, and less than 15 per cent received baseline screening. Young adults aged 19 and 34 years were the least likely to undergo metabolic monitoring. Being older, having a greater number of chronic conditions, more primary care visits, and visits with the same provider resulted in better metabolic monitoring. Most screening tests were ordered by family physicians. Overall, screening practices worsened slightly in the post-pandemic period.

**Conclusion:**

Metabolic monitoring following treatment with atypical antipsychotics was lowest among young adults and adults newly initiated on atypical antipsychotics. Frequent primary care visits and continuity with the same providers resulted in improved screening.

## Résumé

**Objectif:**

Nous examinons la fréquence de mise en application des pratiques de dépistage et de surveillance recommandées pour détecter les changements métaboliques après l’administration d’un traitement par antipsychotiques atypiques en comparant des données des périodes pré-pandémique et post-pandémique. Nous avons aussi comparé les caractéristiques des adultes qui ont fait l’objet de tests de dépistage à celles des adultes qui n’en ont pas fait l’objet pour définir les populations mal desservies.

**Méthodes:**

Nous utilisons les données administratives de la Colombie-Britannique (Canada) fondées sur la population datant de 2018 à 2023 pour présenter de l’information sur les caractéristiques des adultes ayant été soumis ou non au test de laboratoire dans les délais recommandés. Afin d’établir les tests de dépistage initiaux, la cohorte a été stratifiée en groupes de patients ayant déjà reçu un antipsychotique et de patients n’en ayant jamais reçu. Nous décrivons le nombre et la proportion de chaque test de laboratoire dont les patients ont fait l’objet, ainsi que la spécialité du fournisseur ayant soumis la demande au cours des différentes périodes.

**Résultats:**

Plus du tiers de la population n’a reçu aucun des tests de dépistage recommandés, et moins de 15 % des patients de la population ont fait l’objet d’un dépistage initial. Les jeunes adultes âgés de 19 à 34 ans étaient les moins susceptibles de faire l’objet d’une surveillance de leur métabolisme. L’âge plus avancé, la présence d’un nombre accru de maladies chroniques, un nombre accru de consultations en soins primaires et le fait de consulter le même fournisseur ont donné lieu à une meilleure surveillance du métabolisme. La plupart des tests de dépistage ont été demandés par les médecins de famille. Dans l’ensemble, les pratiques de dépistage se sont légèrement détériorées durant la période post-pandémique.

**Conclusion:**

La plus faible surveillance du métabolisme après le traitement par des antipsychotiques atypiques a été observée chez les jeunes adultes et chez les adultes chez lesquels l’instauration du traitement par des antipsychotiques atypiques était récente. Les consultations fréquentes en soins primaires et auprès des mêmes fournisseurs ont donné lieu à un meilleur dépistage.

## Introduction

Clinical practice guidelines recommend atypical (or second- and third-generation) antipsychotics as the first-line treatment for schizophrenia and bipolar disorders. Atypical antipsychotics are preferred over first-generation antipsychotics due to their lower risk of extrapyramidal (movement-related) side effects.^1^ Accordingly, atypical antipsychotic prescription has been increasing in Western countries in recent decades.^2^ Off-label prescribing has also increased across Canada.^3,4^ Between 1996 and 2011, atypical antipsychotic dispensing increased 18.1 fold (from 0.33 to 5.98 per 1000 population) in British Columbian youth under 18 years of age, where the most common diagnoses associated with prescriptions were anxiety disorders, depressive disorders, developmental delays, and hyperkinetic syndrome.^5^ A nationwide study found a 300 per cent increase in the dispensed prescription of the atypical antipsychotic quetiapine between 2005 and 2012, with a two-fold increase for treating anxiety disorders and a 10-fold increase for treating sleep disorders.^6^

However, atypical antipsychotics are associated with adverse effects, including liver enzyme abnormalities,^7^ metabolic abnormalities (including weight gain and lipid and glucose dysregulation leading to metabolic syndrome),^8^ and hypertension and obesity in adults.^9^ This is compounded by the fact that individuals with bipolar disorders and schizophrenia have a higher risk of developing metabolic syndrome^10,11^ due to risk factors such as a sedentary lifestyle, poor diet,^12^ and genetic predispositions.^13^ Use of antipsychotics can also increase the risk for cardiovascular disease.^14^ A large-scale meta-analysis reported that individuals with bipolar disorders, major depressive disorder, and schizophrenia have a 53 per cent higher risk of having cardiovascular disease and an 85 per cent higher risk of dying from cardiovascular disease than the matched general population.^15^ Most premature deaths among people with schizophrenia and bipolar disorders are from physical illnesses,^16^ emphasizing the critical need for preventive interventions and increased attention for this population.

Early detection and intervention can help address metabolic syndrome and prevent the development of diabetes, hypertension, heart disease, stroke,^17–19^ and premature mortality.^20^ Clinical practice guidelines recommend routine monitoring of metabolic indicators in individuals prescribed atypical antipsychotics before treatment initiation to establish baseline values, at 3 months to allow adequate observation of treatment-related effects, and annually for the duration of the treatment.^21,22^ However, adherence to these monitoring recommendations remains low.^23–25^ While interventions at the providers,^26^ patients,^27,28^ and system levels^29,30^ have been shown to improve monitoring practices, a systematic review including studies from North America, the United Kingdom, Australia, and East Asia found that about one-third of patients remain unscreened.^26^

Some studies have found that screening for metabolic syndromes varied by patient and provider characteristics.^31,32^ Although there is interest in improving monitoring practices,^33^ very little is known about the characteristics of adults who do not receive recommended monitoring. Examining these characteristics can help identify missed patient populations and inform efforts to improve the safety of antipsychotic use. While two Ontario studies examined monitoring practices among adults,^25,34^ available data in British Columbia (BC) focuses only on children and adolescents.^35^ The COVID-19 pandemic produced lasting changes in health service delivery. Although physician visit volumes in BC returned to near pre-pandemic levels by the fall of 2020, primary care shifted largely to virtual care.^36^ Despite these system-wide changes, no studies have examined how metabolic syndrome screening has been affected in the post-pandemic period. This study uses population-wide administrative data from BC, Canada, to describe the rate of recommended monitoring for the entire adult population who were dispensed atypical antipsychotics and compares the characteristics of adults who received recommended monitoring to those who did not in pre-pandemic and post-pandemic periods. The post-pandemic period is defined here as the phase following the acute COVID-19 health service disruptions (March–October 2020), during which health services gradually returned to pre-pandemic volumes and adapted their delivery models in BC.

## Methodology

### Study Population

We used de-identified population-based health administrative data linked by Population Data BC, which includes data on all British Columbians enrolled in BC's public health insurance under the Medical Services Plan (MSP). For the pre-pandemic period, we included all adults aged 19 years and older who were dispensed atypical antipsychotics in community pharmacies from April 1, 2018 to March 31, 2019 (pre-pandemic accrual period) who were registered for MSP for at least 275 days in the 1 year following their first atypical antipsychotic dispensing within the pre-pandemic accrual period. For the post-pandemic period, we included all adults aged 19 years of age and older who were dispensed atypical antipsychotics in community pharmacies from April 1, 2021 to March 31, 2022 (post-pandemic accrual period) and were registered for MSP for at least 275 days in the 1 year following their first atypical antipsychotic dispensing within the post-pandemic accrual period. We excluded people who were in long-term care facilities during the study years or died within the 1-year follow-up period, during which we would expect follow-up lab tests. We also excluded people who received only one prescription or one refill within 30 days of their first prescription or who did not have a follow-up dispensing within 180 days of their first dispensing during the study period, as we would not expect follow-up lab tests.

### Study Variables

#### Population Characteristics

People's age and administrative sex were determined from the MSP registration file. The option for gender is limited to “M” and “F” on the MSP registration file. As such, it cannot be determined whether legal sex, sex assigned at birth, or gender is reflected.

We determined neighbourhood income quintiles using census dissemination areas of residence based on version 7E (2018/19) and version 8A (2021/22) of the Postal Code Conversion File (PCCF+). We categorized patients’ residences as urban, other urban, and rural using Statistics Canada's Statistical Area Classification Metropolitan Influences Zones. We assigned regional health authorities using postal codes. Using the PharmaNet database, we identified people covered under the low-income drug plan (BC Income Assistance Plan C) and the psychiatric medication drug plan (Plan G) during their 1-year follow-up period.

We categorized people as immigrants if they had a record in the Immigration, Refugee and Citizenship Canada (IRCC) data and people without a record in the IRCC data as non-immigrants. However, the IRCC data does not capture undocumented migrants, people with temporary work permits, international students, and refugee claimants.

We looked back 3 years from the date of the first dispensing in the accrual periods to capture the number of Charlson comorbid conditions, the Charlson comorbidity index,^37^ and mental illness diagnosis. We identified people who received services for mental illnesses using ICD-9 and ICD-10 codes. Using a validated algorithm, we categorized people who were treated for “*schizophrenia, schizoaffective disorders, and psychotic disorders not otherwise specified*.”^38^ People who did not meet the criteria for the validated algorithm for schizophrenia and related disorders but received one or more visit(s) in outpatient or inpatient for any condition with psychosis in the previous 3 years were categorized as having “*Other conditions with psychosis*,” which included substance-induced psychosis and conditions treated with antipsychotics as a mood stabilizer, such as bipolar disorders All individuals who were not treated for the conditions in these two categories in the previous 2 years were categorized as having an “*other mental or health condition*” (see Table S1 in Supplemental material for the complete list of conditions and ICD-9 and ICD-10 codes).

We captured the number of primary care visits with family physicians, general physicians, and nurse practitioners based on unique provider-patient-date combinations that occurred in an outpatient setting (excluding hospital visits and emergency visits) during the accrual periods and the proportion of these visits in the 2 years preceding them. We used the Bice-Bozeman Continuity of Care Index (COCI)^39^ and the usual provider of care index to measure continuity of care with a primary care provider in the accrual periods.

#### Atypical Antipsychotics

Atypical antipsychotics were identified using the WHO's Anatomical Therapeutic Chemical (ATC) classification system.^40^ We then used the ATC codes to pull all Drug Identification Numbers (DINs) from Health Canada's Drug Product Database and BC-specific Product Identification Numbers (PINs) from the Government of British Columbia's website for combination drugs to identify all atypical antipsychotics prescribed in BC. This study included the following atypical antipsychotics: ziprasidone, lurasidone, olanzapine, quetiapine, asenapine, olanzapine and samidorphan, amisulpride, risperidone, paliperidone, pimavanserin, aripiprazole, cariprazine, and brexpiprazole. We excluded clozapine from this study as it requires rigorous monitoring and frequent testing that is different from other atypical antipsychotics.^41^

#### Lab Tests for Monitoring Metabolic Changes

Some clinical practice guidelines recommend baseline testing prior to initiating pharmacotherapy, follow-up testing at least 3 months following initiation of atypical antipsychotics to assess metabolic changes, and then once every 12 months.^22,42^ To capture baseline and follow-up testing, we grouped people who were not hospitalized for a mental illness in the past 6 months and were dispensed atypical antipsychotics in the community for the first time in at least 6 months in the naïve cohort and grouped people who were dispensed atypical antipsychotics continuously or with less than a 6-month gap between dispensed atypical antipsychotics in the community in the non-naïve cohort during the accrual periods (pre-pandemic period: 2018/19; post-pandemic period 2021/22). Baseline testing is clinically indicated with a 6-month period free of antipsychotics.^43^ People who received atypical antipsychotics in the community for the first time in at least 6 months but were hospitalized for a mental illness within the past 6 months were grouped in the non-naïve cohort, as we cannot capture antipsychotics dispensed during hospitalization.

**Figure 1.**
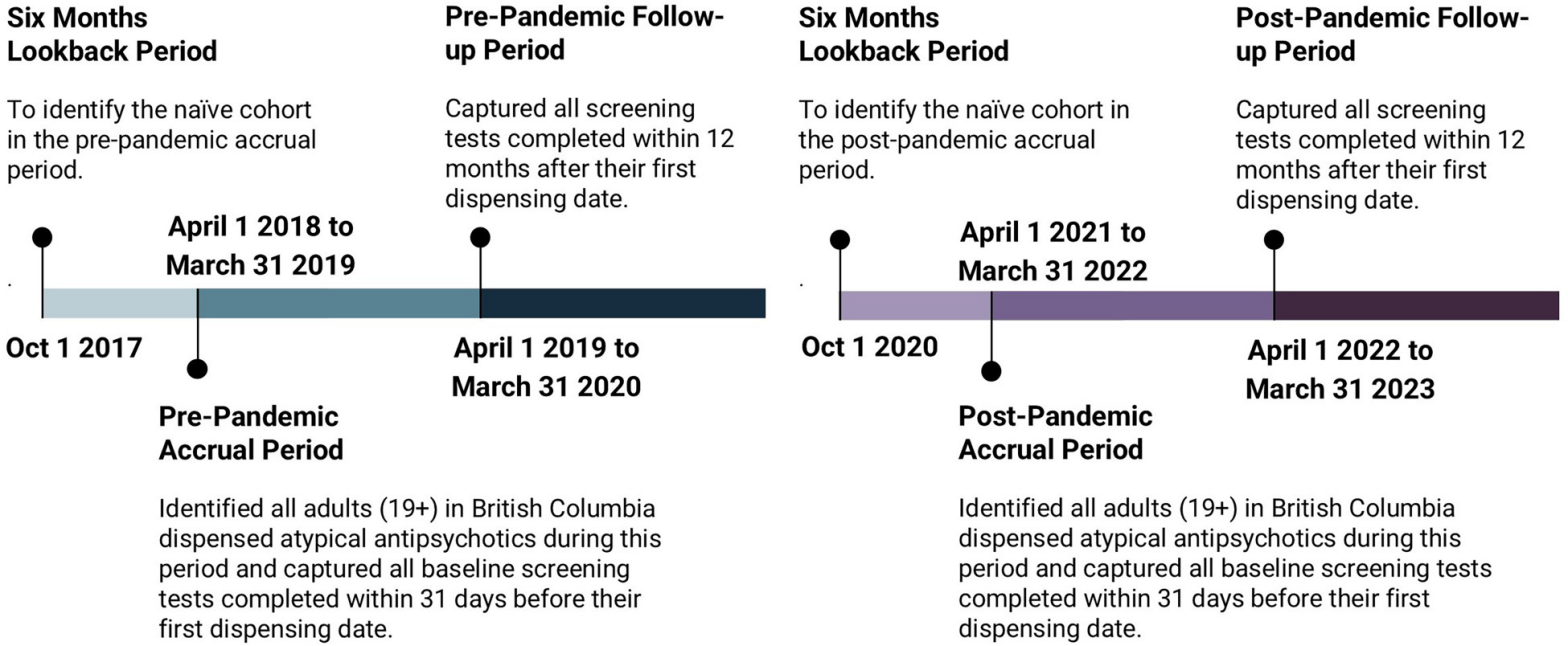
Timeline to identify study population and capture screening tests for both pre- and post-pandemic periods.

We captured lab tests recommended to monitor metabolic changes from atypical antipsychotics,^22^ which include tests to capture liver profile: aspartate aminotransferase (AST), glutamyl transpeptidase (GGT or GTP), and alanine transaminase/aminotransferase (ALT); lipid profile: triglycerides, high-density lipoprotein (HDL), and total cholesterol; blood glucose: glycated haemoglobin (A1c) and fasting glucose; complete blood count (CBC); and prolactin. For the naïve cohort, we captured lab tests performed in three time periods: baseline lab tests performed within 31 days prior to their first prescription,^44^ follow-up lab tests performed within 3 months following the first prescription, and follow-up lab tests performed from 3 to 12 months after the first prescription. For the non-naïve cohort, we captured follow-up lab tests performed at any time within 12 months following their first prescription (see Figure 1).

We identified the clinical specialty of the healthcare providers who ordered each lab test based on their billing claims. Family physicians billed with Claim Specialty “00”, psychiatrists billed with Claim Specialty “03”, and nurse practitioners billed with Claim Specialty “87”.

### Statistical Analysis

We performed descriptive statistics to compare the demographic characteristics of patients who received any of the monitoring lab tests with those who did not receive any test within the recommended periods for naïve and non-naïve cohorts. We describe the number and percentage of each lab test and the provider's specialty ordering each lab test within different periods. We present counts, percentages, proportions, means, standard deviations, medians, and standardized differences.^45^ All statistical analyses were performed using SAS software, Version 9.4 of the SAS system.^46^

## Results

### Characteristics of People Who Received Atypical Antipsychotics

We identified 82,679 patients in the pre-pandemic period (2018/19) and 97,502 patients in the post-pandemic period (2021/22) who received more than one refill of atypical antipsychotics (Table 1) after excluding patients who were in long-term care [n = 28,531], patients who only received one refill within 30 days or did not have a follow-up dispensing within 180 days of their first prescription [n = 42,306], patients who died in the follow-up years [n = 1,179]. Population characteristics were comparable in the two study periods. Approximately half (52.0% in pre-pandemic and 53.4% post-pandemic) were female. In the year following their first prescription, 39.0 and 35.4 per cent were dispensed atypical antipsychotics under the income assistance drug plan (Plan C) and 20.4 and 19.0 per cent were dispensed atypical antipsychotics under the psychiatric medicine plan (Plan G) for the pre-pandemic and post-pandemic periods, respectively.

**Table 1.** Characteristics of Adults on Atypical Antipsychotics Who Received or Did Not Receive Monitoring Lab Tests in British Columbia.

	Pre-Pandemic (2018/19 and 2019/20)				Post-Pandemic (2021/22 and 2022/23)			
Characteristics	Received any Test	Did Not Receive any Test	Total	^a^	Received any Test	Did Not Receive any Test	Total	^a^
Overall, N (%)	54,790 (66.27)	27,889 (33.73)	82,679 (100)	.	59,590 (61.12)	37,912 (38.88)	97,502 (100)	.
Age group, N (%)								
19–34	10,163 (18.6)	8,839 (31.7)	19,002 (23.0)	.44	11,312 (19.0)	12,365 (32.6)	23,677 (24.3)	.44
35–44	8,089 (14.8)	5,753 (20.6)	13,842 (16.7)	.	8,827 (14.8)	7,622 (20.1)	16,449 (16.9)	.
45–64	23,764 (43.4)	10,041 (36.0)	33,805 (40.9)	.	23,814 (40.0)	12,811 (33.8)	36,625 (37.6)	.
65+	12,774 (23.3)	3,256 (11.7)	16,030 (19.4)	.	15,637 (26.2)	5,114 (13.5)	20,751 (21.3)	.
Administrative sex, N (%)								
F	29,948 (54.7)	13,048 (46.8)	42,996 (52.0)	.16	33,596 (56.4)	18,512 (48.8)	52,108 (53.4)	.15
M	24,842 (45.3)	14,841 (53.2)	39,683 (48.0)	.	25,994 (43.6)	19,400 (51.2)	45,394 (46.6)	.
Immigration status N (%)								
Immigrant	6,667 (12.2)	2,563 (9.2)	9,230 (11.2)	.10	8,171 (13.7)	3,994 (10.5)	12,165 (12.5)	.10
Non-immigrant	48,123 (87.8)	25,326 (90.8)	73,449 (88.8)	.	51,419 (86.3)	33,918 (89.5)	85,337 (87.5)	.
Rurality, N (%)								
1- Metro	35,717 (65.2)	17,769 (63.7)	53,486 (64.7)	.04	43,952 (73.8)	27,021 (71.3)	70,973 (72.8)	.05
2- Other urban	12,645 (23.1)	6,689 (24.0)	19,334 (23.4)	.	9,364 (15.7)	6,455 (17.0)	15,819 (16.2)	.
3- Rural	6,021 (11.0)	3,098 (11.1)	9,119 (11.0)	.	5,880 (9.9)	4,059 (10.7)	9,939 (10.2)	.
Unknown	407 (0.7)	333 (1.2)	740 (0.9)	.	394 (0.7)	377 (1.0)	771 (0.8)	.
Neighbourhood income quintile, N (%)								
Q1 (lowest)	17,212 (31.4)	8,821 (31.6)	26,033 (31.5)	.04	17,589 (29.5)	11,319 (29.9)	28,908 (29.7)	.00
Q2	11,663 (21.3)	5,686 (20.4)	17,349 (21.0)	.	12,846 (21.6)	8,199 (21.6)	21,045 (21.6)	.
Q3	9,857 (18.0)	4,961 (17.8)	14,818 (17.9)	.	10,841 (18.2)	6,824 (18.0)	17,665 (18.1)	.
Q4	8,353 (15.3)	4,327 (15.5)	12,680 (15.3)	.	9,482 (15.9)	5,982 (15.8)	15,464 (15.9)	.
Q5 (highest)	7,288 (13.3)	3,757 (13.5)	11,045 (13.4)	.	8,400 (14.1)	5,172 (13.6)	13,572 (13.9)	.
Unknown	417 (0.8)	337 (1.2)	754 (0.9)	.	432 (0.7)	416 (1.1)	848 (0.9)	.
Health authority, N (%)								
Interior	9,530 (17.4)	5,213 (18.7)	14,743 (17.8)	.06	10,415 (17.5)	7,381 (19.5)	17,796 (18.3)	.07
Fraser	18,911 (34.5)	9,773 (35.0)	28,684 (34.7)	.	20,755 (34.8)	12,871 (34.0)	33,626 (34.5)	.
Vancouver Coastal	12,304 (22.5)	5,897 (21.1)	18,201 (22.0)	.	13,349 (22.4)	7,472 (19.7)	20,821 (21.4)	.
Island	10,285 (18.8)	4,967 (17.8)	15,252 (18.5)	.	11,012 (18.5)	7,212 (19.0)	18,224 (18.7)	.
Northern	3,713 (6.8)	1,990 (7.1)	5,703 (6.9)	.	4,008 (6.7)	2,925 (7.7)	6,933 (7.1)	.
Unknown	47 (0.1)	49 (0.2)	96 (0.1)	.	51 (0.1)	51 (0.1)	102 (0.1)	.
PCare Income Assistance Plan C in 1-year follow-up, N (%)								
N	34,229 (62.5)	16,216 (58.1)	50,445 (61.0)	.09	39,656 (66.6)	23,323 (61.5)	62,979 (64.6)	.10
Y	20,561 (37.5)	11,673 (41.9)	32,234 (39.0)	.	19,934 (33.5)	14,589 (38.5)	34,523 (35.4)	.
PCare Psychiatric Medicine Plan G in 1-year follow-up, N (%)								
N	43,703 (79.8)	22,084 (79.2)	65,787 (79.6)	.01	48,486 (81.4)	30,507 (80.5)	78,993 (81.0)	.02
Y	11,087 (20.2)	5,805 (20.8)	16,892 (20.4)	.	11,104 (18.6)	7,405 (19.5)	18,509 (19.0)	.
# Charlson comorbidities (3-year lookback), N (%)								
0	23,071 (42.1)	17,833 (63.9)	40,904 (49.5)	.45	24,721 (41.5)	24,007 (63.3)	48,728 (50.0)	.45
1+	31,719 (57.9)	10,056 (36.1)	41,775 (50.5)	.	34,869 (58.5)	13,905 (36.7)	48,774 (50.0)	.
# of Charlson comorbidities (3-year lookback)								
Mean ± SD	1.08 ± 1.27	0.54 ± 0.90	0.90 ± 1.19	.48	1.11 ± 1.29	0.56 ± 0.92	0.90 ± 1.19	.49
Median (IQR)	1 (0–2)	0 (0–1)	1 (0–1)	.50	1 (0–2)	0 (0–1)	1 (0–1)	.51
Diagnosis criteria met in 3-year lookback, N (%)								
Schizophrenia, schizoaffective disorders, and psychotic disorders not otherwise specified	11,895 (21.7)	6,073 (21.8)	17,968 (21.7)	.09	11,754 (19.7)	8,100 (21.4)	19,854 (20.4)	.06
Other conditions with psychosis	20,511 (37.4)	9,327 (33.4)	29,838 (36.1)	.	21,452 (36.0)	12,533 (33.1)	33,985 (34.9)	.
Other mental or health conditions	22,384 (40.9)	12,489 (44.8)	34,873 (42.2)	.	26,384 (44.3)	17,279 (45.6)	43,663 (44.8)	.
# outpatient visits with family physicians and nurse practitioners								
Mean ± SD	11.62 ± 11.93	8.56 ± 12.25	10.59 ± 12.13	.25	12.96 ± 12.17	9.23 ± 12.32	11.51 ± 12.36	.30
Median (IQR)	8 (5–14)	5 (2–9)	7 (4–13)	.54	10 (6–16)	6 (3–11)	8 (4–14)	.58
Usual provider of care index								
Mean ± SD	0.77 ± 0.23	0.76 ± 0.25	0.76 ± 0.23	.05	0.80 ± 0.22	0.79 ± 0.23	0.80 ± 0.22	.03
Median (IQR)	0.82 (0.6–1)	0.81 (0.5–1)	0.82 (0.58–1)	.01	0.87 (0.67–1)	0.88 (0.63–1)	0.88 (0.64–1)	.02
Continuity of care index								
Mean ± SD	0.62 ± 0.32	0.57 ± 0.36	0.61 ± 0.33	.15	0.68 ± 0.30	0.64 ± 0.35	0.67 ± 0.32	.11
Median (IQR)	0.64 (0.36–1)	0.58 (0.27–1)	0.62 (0.33–1)	.14	0.73 (0.43–1)	0.70 (0.33–1)	0.71 (0.4–1)	.08
% of family physicians and nurse practitioners outpatient visits with known provider								
Mean ± SD	82.26 ± 22.71	74.75 ± 30.21	79.85 ± 25.60	.28	85.75 ± 20.49	79.57 ± 28.12	83.46 ± 23.80	.25
Median (IQR)	88.89 (75–100)	85.71 (60–100)	88.46 (71.43–100)	.17	92.31 (80–100)	90.91 (70–100)	92.31 (77.78–100)	.12

*Note*. ^a^standardized difference; Q = income quintile; SD = standard deviation; IQR = interquartile range; PCare = PharmaCare medication plan; Plan G = Psychiatric medication plan; Plan C = income assistance.

About a fifth of the study population met the criteria for the validated algorithm for *schizophrenia, schizoaffective, and psychotic disorders not otherwise specified* (21.7% pre-pandemic and 20.4% post-pandemic) and about 35 per cent for a *condition with psychosis* (36.1% in pre-pandemic and 34.9% in post-pandemic). Over 40 per cent (42.2% in pre-pandemic and 44.8% in post-pandemic) of those dispensed atypical antipsychotics were not treated for a condition with psychosis in the previous 3 years. About half had at least one Charlson comorbidity (50.5% pre-pandemic and 50.0% post-pandemic).

### Comparison of People Who Received any Lab Test With People Who Did Not

Overall, 54,790 (66.27%) and 59,590 (61.12%) received at least one of the recommended tests (including baseline tests) to detect metabolic changes during the pre-pandemic and post-pandemic periods, respectively (Table 1). People who received any lab tests were older, had at least one Charlson comorbidity condition and had a higher average number of Charlson comorbidity conditions than people who did not receive any of the recommended lab tests. They also had a greater number of primary care visits and a higher proportion of visits with the same primary care provider than those not tested. This pattern of differences was consistent across the pre-pandemic and post-pandemic study periods (Table 1).

### Baseline and Follow-up Monitoring

Among the 16,541 antipsychotic-naïve patients in pre-pandemic and the 18,963 in post-pandemic periods, 14.26 and 12.66 per cent took any recommended baseline lab tests, respectively (Table 2). Regarding follow-up monitoring, 53.84 and 50.15 per cent of the antipsychotic-naïve patients were tested at least 3 months following initiation, as clinically recommended, while 32.05 and 29.15 per cent were tested before the recommended 3-month period during pre- and post-pandemic periods, respectively. Among the 66,138 non-naïve patients during the pre-pandemic and 78,539 non-naïve people during the post-pandemic periods, 66.88 and 61.28 per cent underwent any recommended follow-up lab tests at least once in the follow-up year, respectively.

**Table 2.** Monitoring Lab Testing in Naïve and Non-Naïve Cohorts Receiving Atypical Antipsychotics and the Specialty of Provider Ordering the Test in British Columbia.

		Pre-Pandemic (2018/19 and 2019/20)				Post-Pandemic (2021/22 and 2022/23)			
		Naïve Cohort, N (%)			Non-Naïve Cohort, N (%)	Naïve Cohort, N (%)			Non-Naïve Cohort, N (%)
		Baseline (Within 31 Days)	Follow-up (0–89 Days)	Follow-up After 3 Months	Follow-up (Once a Year)	Baseline (Within 31 Days)	Follow-up (0–89 Days)	Follow-up After 3 Months	Follow-up (Once a Year)
Number of people, N		16,541	16,541	16,541	66,138	18,963	18,963	18,963	78,539
Any lab test (listed below)		2,358 (14.26)	5,302 (32.05)	8,905 (53.84)	44,230 (66.88)	2,401 (12.66)	5,528 (29.15)	9,510 (50.15)	48,128 (61.28)
Liver profile	AST	545 (3.29)	1,285 (7.77)	2,418 (14.62)	12,847 (19.42)	534 (2.82)	1,317 (6.95)	2,581 (13.61)	13,999 (17.82)
	ALT	1,352 (8.17)	3,264 (19.73)	6,009 (36.33)	31,650 (47.85)	1,459 (7.69)	3,531 (18.62)	6,632 (34.97)	35,255 (44.89)
	GTP	886 (5.36)	2,264 (13.69)	4,225 (25.54)	22,710 (34.34)	920 (4.85)	2,376 (12.53)	4,658 (24.56)	25,111 (31.97)
Lipid profile	Triglycerides	624 (3.77)	1,801 (10.89)	3,872 (23.41)	24,004 (36.29)	750 (3.96)	2,227 (11.74)	4,641 (24.47)	28,433 (36.20)
	HDL	664 (4.01)	1,895 (11.46)	4,060 (24.55)	25,239 (38.16)	784 (4.13)	2,333 (12.30)	4,861 (25.63)	29,651 (37.75)
	Total Cholesterol	667 (4.03)	1,896 (11.46)	4,064 (24.57)	25,261 (38.19)	785 (4.14)	2,336 (12.32)	4,865 (25.66)	29,668 (37.77)
Blood glucose	Haemoglobin A1c	977 (5.91)	2,364 (14.29)	5,021 (30.35)	27,534 (41.63)	1,154 (6.09)	3,067 (16.17)	6,128 (32.32)	34,441 (43.85)
	Fasting glucose	831 (5.02)	2,017 (12.19)	4,061 (24.55)	23,955 (36.22)	800 (4.22)	2,086 (11.00)	4,185 (22.07)	24,263 (30.89)
Blood profile	CBC	2,032 (12.28)	4,640 (28.05)	8,155 (49.30)	40,423 (61.12)	2,105 (11.10)	4,882 (25.74)	8,786 (46.33)	44,312 (56.42)
Prolactin	Prolactin	43 (0.26)	156 (0.94)	373 (2.26)	2,159 (3.26)	57 (0.30)	185 (0.98)	447 (2.36)	2,338 (2.98)
For those who received a lab test, specialty of provider ordering lab test(s)									
Family physician		1,692 (71.76)	3,820 (72.05)	7,149 (80.28)	35,378 (79.99)	1,646 (68.55)	3,996 (72.29)	7,427 (78.10)	37,687 (78.31)
Psychiatrist		187 (7.93)	594 (11.20)	940 (10.56)	7,806 (17.65)	131 (5.46)	446 (8.07)	692 (7.28)	6,544 (13.60)
Nurse practitioner		76 (3.22)	225 (4.24)	319 (3.58)	1,824 (4.12)	102 (4.25)	236 (4.27)	497 (5.23)	2,542 (5.28)
Other practitioners^a^		513 (21.76)	1,182 (22.29)	2,271 (25.50)	11,129 (25.16)	612 (25.49)	1,374 (24.86)	2,682 (28.20)	12,777 (26.55)

*Note*. A1c = glycated haemoglobin; ALT = Alanine transaminase/aminotransferase; AST = Aspartate aminotransferase; CBC = complete blood count; GTP = Glutamyl transpeptidase; HDL = high-density lipoprotein.

^a^ The “other practitioners” category also includes practitioners with unidentified specialties in the data, which may include family physicians, psychiatrists, and nurse practitioners.

Across both study periods, both naïve and non-naïve patients most frequently completed tests for CBC, followed by ALT and A1c. Recommended lab tests for the lipid panel (triglycerides, HDL, and total cholesterol) were completed by around 4 per cent of naïve people at baseline and about 25 per cent of non-naïve people at follow-up after 3 months during both study periods. About a third of non-naïve people had a follow-up lipid panel test done (Table 2).

The majority of the completed lab tests, ranging from 68.55 to 80.28 per cent, were ordered by a family physician, followed by other practitioners, psychiatrists, and nurse practitioners. This pattern was consistent across all follow-up timeframes and study periods (Table 2).

## Discussion

Our population-based study revealed six key findings: (1) over a third of the population did not receive any of the recommended lab tests for monitoring metabolic changes at baseline or follow-up; (2) baseline lab tests for the antipsychotic-naïve population were infrequent (<15%); (3) recommended lab tests were lowest among young adults aged 19 to 34 years; (4) people who completed lab tests had more chronic comorbid conditions, primary care visits, and visits with the same provider; (5) family physicians ordered the majority of lab tests; and (6) patterns of lab test completion did not differ by rurality, neighbourhood income, or Health Authority. However, overall rates of monitoring decreased slightly in the post-pandemic period.

The finding that about one-third of people in British Columbia who were dispensed atypical antipsychotics did not complete any recommended lab tests is similar to rates found in systematic reviews, though the rates varied across these studies.^26,47^ The finding that <15% of the antipsychotic-naïve people received any of the recommended lab tests at baseline is also in keeping with studies from the United States^29^ and Ireland.^48^ Baseline tests may be useful for comparing metabolic changes associated with initiating antipsychotics, and the same tests are also relevant for other medication classes prescribed to treat chronic conditions. Additionally, results from baseline tests may be used to screen for early indications of conditions prevalent in this population and could inform practitioners in selecting medications to minimize risks and complications.^42^

We found that completed lab tests for metabolic dysfunction were lowest among young adults, consistent with findings from other jurisdictions.^23,49^ Young adults treated with antipsychotics could experience greater benefit from regular screening and prevention accruing over the life course. Conversely, we found that being older and having more comorbidities are related to receiving more lab tests. These findings are consistent with Fisher and colleagues, who examined factors associated with screening test.^32^ Frequent lab tests in older adults and adults with comorbidities may be due to health reasons unrelated to monitoring for adverse effects from antipsychotics, as the same lab tests are used to screen for and monitor several chronic conditions.

Our results showed that the rate of recommended lab tests to monitor metabolic syndrome dropped slightly in the post-pandemic period. This finding is consistent with the literature showing that routine diabetes monitoring,^50^ laboratory testing for chronic disease surveillance,^51^ and cancer screening have not returned to pre-pandemic levels even 2 years after the onset of the pandemic.^52^ While the shift to virtual care limits physicians’ ability to physically examining their patients for metabolic syndrome (i.e., body mass index [BMI] and blood pressure), there is limited evidence on whether virtual care influences the rate of lab tests. One Ontario study reported that individuals with higher levels of virtual-care use underwent more lab testing than those with lower virtual-care use, and these individuals consistently demonstrated high service utilization before and after the pandemic, suggesting that the shift to virtual care did not impede screening in high-service users.^53^ Evidence suggests that the disruption to health service capacity during the peak of the COVID-19 pandemic—including the suspension or scaling back of many screening and monitoring activities to divert efforts toward COVID-19 response—created substantial backlogs and long-term ripple effects that likely worsened prevention services.^54^

Our results showed that monitoring patterns did not differ by rurality, despite studies indicating that rural regions in Canada tend to have lower access to outpatient primary care services.^55,56^

Our study revealed that family physicians ordered the majority of lab tests completed by patients, and that higher interactions with primary care services and the same primary care provider resulted in more lab tests. Currently, Canadians are experiencing limited access to family physicians due to population growth, an aging population with medical complexities, and physicians retiring or increasingly practising in settings outside primary care.^57^ For instance, about one in 10 young adults with psychosis in Ontario does not have regular access to a family physician.^58^ Further study could assess the relationship between access to primary care and antipsychotic monitoring.

However, increasing access to consistent primary care services may not be sufficient to address concerns about monitoring for side effects of antipsychotic medications. Two systematic reviews on the perspectives of providers and patients^59,60^ highlight other barriers. Healthcare providers reported that poor collaboration between health professionals, fragmented health systems, lack of knowledge of metabolic side effects, and time constraints are common barriers to monitoring the metabolic side effects of antipsychotic medications. Meanwhile, patients reported that disability from their mental illness(es) and chronic condition(s), lack of knowledge of metabolic side effects, and poor relationship and trust with their providers are common barriers.^59,60^ Additionally, fear and anxiety related to needles and pain have been documented as being patient-level barriers to receiving required blood tests.^61,62^

Interventions targeting provider-based barriers included education training sessions and electronic prompts to remind physicians when a screening was due for their patients. Interventions targeting patient-level barriers included education and empowerment strategies to encourage patients to request screening.^26^ However, most interventions were designed to address system-level barriers, such as strategies to encourage physician adherence to guidelines, audits of metabolic monitoring practices, assessments of workplace barriers, and promoting collaboration between primary care and mental health services.^26^ Shin and colleagues’ meta-analyses of interventions delivered in inpatient settings revealed that interventions targeting both providers and patients were the most successful at increasing metabolic monitoring practices.^63^ In BC, educating community mental health teams using training workshops and a handbook on metabolic monitoring improved monitoring for children and adolescents.^64^

Our study had several strengths. We were able to access population-wide administrative data that captures over five million publicly insured British Columbians. This dataset includes all prescriptions dispensed in community pharmacies as well as hospital outpatient pharmacies, allowing us to closely approximate true rates of atypical antipsychotics dispensed and completed lab tests within the provincial population. Furthermore, administrative data allowed us to describe and compare numerous demographic characteristics of our study population that have not been typically explored in previous studies on monitoring practices for antipsychotics, including neighbourhood income quintile, urban versus rural areas, Charlson comorbidities, immigration status, and primary care service use and continuity of care.

In terms of limitations, we cannot identify the health concerns associated with the lab tests completed in the administrative data. Therefore, it is possible that the lab tests were for health concerns unrelated to the use of antipsychotics. This could explain why older adults—who are more likely to experience chronic conditions—were overrepresented among those who completed recommended lab tests. Additionally, administrative data cannot capture other key clinically recommended parameters to monitor metabolic changes, such as waist circumference and BMI. As administrative data capture only lab tests that patients have completed, this study could not account for all lab requisitions ordered by providers, nor distinguish between patient- and provider-level contributors to poor monitoring rates. This limitation should be considered when interpreting our results. Lastly, administrative data does not include lab tests and antipsychotics dispensed while people are hospitalized. Patients hospitalized during the lookback period were assumed to be non-naïve since they could have received atypical antipsychotics while hospitalized. However, sensitivity analysis accounting for people who were hospitalized in the naïve cohort did not change our findings.

## Conclusion

We found that about one-third of all people who received atypical antipsychotics do not undergo any of the recommended lab tests to monitor for metabolic syndrome, and young adults were the least likely to be screened. Monitoring rates were also poorer among people newly initiated on atypical antipsychotics compared to those with longer-term use. In contrast, increased interactions with primary care resulted in higher rates of recommended lab tests. Monitoring rates were slightly lower than pre-pandemic levels even after health service volumes largely recovered following COVID-19 disruptions. Future intervention strategies should focus on improving monitoring practices, particularly for younger adults and people new to antipsychotics.

## Data Availability

Access to data provided by the Data Stewards is subject to approval but can be requested for research projects through the Data Stewards or their designated service providers. The following data sets were used in this study: Consolidation file, Medical Services Plan (MSP), PharmaNet, Vital Events Deaths, IRCC Permanent Resident Database,^65^ BC Mental Health & Substance Use Services,^66^ Discharge Abstract Database, and National Ambulatory Care Reporting System. You can find further information regarding these data sets by visiting the PopData project webpage at: (https://my.popdata.bc.ca/project_listings/22-081/). All inferences, opinions, and conclusions drawn in this publication are those of the authors, and do not reflect the opinions or policies of the Data Steward(s).

## Supplemental Material

sj-docx-1-cpa-10.1177_07067437261420884 - Supplemental material for Monitoring Rates for Metabolic Syndrome in Adults Treated With Atypical Antipsychotics: A Population-Based Study in British Columbia: Fréquence de la surveillance du syndrome métabolique chez les adultes traités par des antipsychotiques atypiques : étude fondée sur la population menée en Colombie-Britannique Supplemental material, sj-docx-1-cpa-10.1177_07067437261420884 for Monitoring Rates for Metabolic Syndrome in Adults Treated With Atypical Antipsychotics: A Population-Based Study in British Columbia: Fréquence de la surveillance du syndrome métabolique chez les adultes traités par des antipsychotiques atypiques : étude fondée sur la population menée en Colombie-Britannique by Ridhwana Kaoser, M. Ruth Lavergne, Sandra Peterson, Agnes Grudniewicz, Helen Thai, Lucie Langford, Rita K. McCracken, Sara English and David Rudoler in The Canadian Journal of Psychiatry

## References

[1] Leucht S Corves C Arbter D Engel RR Li C Davis JM . Second-generation versus first-generation antipsychotic drugs for schizophrenia: a meta-analysis. Lancet. 2009;373(9657):31–41. doi:10.1016/S0140-6736(08)61764-X. 19058842

[2] Verdoux H Tournier M Bégaud B . Antipsychotic prescribing trends: a review of pharmaco-epidemiological studies. Acta Psychiatr Scand. 2010;121(1):4–10. doi:10.1111/j.1600-0447.2009.01425.x. 20059452

[3] Alessi-Severini S Biscontri RG Collins DM Sareen J Enns MW . Ten years of antipsychotic prescribing to children: a Canadian population-based study. Can J Psychiatry. 2012;57(1):52–58. doi:10.1177/070674371205700109. 22296959

[4] Rios S Perlman CM Costa A Heckman G Hirdes JP Mitchell L . Antipsychotics and dementia in Canada: a retrospective cross-sectional study of four health sectors. BMC Geriatr. 2017;17(1):244. doi:10.1186/s12877-017-0636-8. 29061129 PMC5651600

[5] Ronsley R Scott D Warburton WP , et al. A population-based study of antipsychotic prescription trends in children and adolescents in British Columbia, from 1996 to 2011. Can J Psychiatry. 2013;58(6):361–369. doi:10.1177/070674371305800608. 23768264

[6] Pringsheim T Gardner DM . Dispensed prescriptions for quetiapine and other second-generation antipsychotics in Canada from 2005 to 2012: a descriptive study. Can Med Assoc Open Access J. 2014;2(4):E225–E232. 10.9778/cmajo.20140009 PMC4251508 25485247

[7] Atasoy N Erdogan A Yalug I , et al. A review of liver function tests during treatment with atypical antipsychotic drugs: a chart review study. Prog Neuropsychopharmacol Biol Psychiatry. 2007;31(6):1255–1260. doi:10.1016/j.pnpbp.2007.05.005. 17600607

[8] Pillinger T McCutcheon RA Vano L , et al. Comparative effects of 18 antipsychotics on metabolic function in patients with schizophrenia, predictors of metabolic dysregulation, and association with psychopathology: a systematic review and network meta-analysis. Lancet Psychiatry. 2020;7(1):64–77. doi:10.1016/S2215-0366(19)30416-X. 31860457 PMC7029416

[9] Hirsch L Yang J Bresee L Jette N Patten S Pringsheim T . Second-generation antipsychotics and metabolicsSide effects: a systematic review of population-based studies. Drug Saf. 2017;40(9):771–781. doi:10.1007/s40264-017-0543-0. 28585153

[10] Mitchell AJ Vancampfort D Sweers K van Winkel R Yu W De Hert M . Prevalence of metabolic syndrome and metabolic abnormalities in schizophrenia and related disorders—a systematic review and meta-analysis. Schizophr Bull. 2013;39(2):306–318. doi:10.1093/schbul/sbr148. 22207632 PMC3576174

[11] Vancampfort D Vansteelandt K Correll CU , et al. Metabolic syndrome and metabolic abnormalities in bipolar disorder: a meta-analysis of prevalence rates and moderators. AJP. 2013;170(3):265–274. doi:10.1176/appi.ajp.2012.12050620. 23361837

[12] Heald A Pendlebury J Anderson S , et al. Lifestyle factors and the metabolic syndrome in schizophrenia: a cross-sectional study. Ann Gen Psychiatry. 2017;16(1):12. doi:10.1186/s12991-017-0134-6. 28289436 PMC5310063

[13] Malan-Müller S Kilian S van den Heuvel LL , et al. A systematic review of genetic variants associated with metabolic syndrome in patients with schizophrenia. Schizophr Res. 2016;170(1):1–17. doi:10.1016/j.schres.2015.11.011. 26621002

[14] De Hert M Detraux J van Winkel R Yu W Correll CU . Metabolic and cardiovascular adverse effects associated with antipsychotic drugs. Nat Rev Endocrinol. 2012;8(2):114–126. doi:10.1038/nrendo.2011.156. 22009159

[15] Correll CU Solmi M Veronese N , et al. Prevalence, incidence and mortality from cardiovascular disease in patients with pooled and specific severe mental illness: a large-scale meta-analysis of 3,211,768 patients and 113,383,368 controls. World Psychiatry. 2017;16(2):163–180. doi:10.1002/wps.20420. 28498599 PMC5428179

[16] Hoang U Goldacre MJ Stewart R . Avoidable mortality in people with schizophrenia or bipolar disorder in England. Acta Psychiatr Scand. 2013;127(3):195–201. doi:10.1111/acps.12045. 23216065

[17] Singh VK Karmani S Malo PK , et al. Impact of lifestyle modification on some components of metabolic syndrome in persons with severe mental disorders: a meta-analysis. Schizophr Res. 2018;202:17–25. doi:10.1016/j.schres.2018.06.066. 30539768

[18] Gurusamy J Gandhi S Damodharan D Ganesan V Palaniappan M . Exercise, diet and educational interventions for metabolic syndrome in persons with schizophrenia: a systematic review. Asian J Psychiatr. 2018;36:73–85. doi:10.1016/j.ajp.2018.06.018. 29990631

[19] Trigueiro AJP Ramirez J Hennessey E Beqiri M . Metabolic syndrome identification in patients treated with second-generation antipsychotic medications. J Psychosoc Nurs Ment Health Serv. 2022;60(8):11–18. doi:10.3928/02793695-20220314-01. 35316124

[20] Laursen TM . Life expectancy among persons with schizophrenia or bipolar affective disorder. Schizophr Res. 2011;131(1):101–104. doi:10.1016/j.schres.2011.06.008. 21741216

[21] Recommendations | Psychosis and schizophrenia in adults: prevention and management | Guidance | NICE. https://www.nice.org.uk/guidance/cg178/chapter/Recommendations (2014, accessed June 11, 2024).

[22] Yatham LN Kennedy SH Parikh SV , et al. Canadian Network for Mood and Anxiety Treatments (CANMAT) and International Society for Bipolar Disorders (ISBD) 2018 guidelines for the management of patients with bipolar disorder. Bipolar Disord. 2018;20(2):97–170. doi:10.1111/bdi.12609. 29536616 PMC5947163

[23] Minjon L van den Ban E Bazelier MT Lalmohamed A Egberts TCG Heerdink ER . Monitoring of adverse drug reaction-related parameters in children, youth, and young adults prescribed antipsychotic drugs by general practitioners. J Child Adolesc Psychopharmacol. 2022;32(1):36–44. doi:10.1089/cap.2021.0026. 34619039 PMC8884168

[24] Azfr Ali RS Jalal Z Paudyal V . Guidelines versus practice in screening and monitoring of cardiometabolic risks in patients taking antipsychotic medications: where do we stand? Gen Psychiatr. 2021;34(4):e100561. doi:10.1136/gpsych-2021-100561. PMC8311327 34396043

[25] Fontaine J Chin E Provencher J-F , et al. Assessing cardiometabolic parameter monitoring in inpatients taking a second-generation antipsychotic: the CAMI-SGA study—a cross-sectional study. BMJ Open. 2022;12(4):e055454. doi:10.1136/bmjopen-2021-055454. PMC9006820 35414553

[26] Melamed OC Wong EN LaChance LR Kanji S Taylor VH . Interventions to improve metabolic risk screening among adult patients taking antipsychotic medication: a systematic review. PS (Wash DC). 2019;70(12):1138–1156. doi:10.1176/appi.ps.201900108. 31522630

[27] Gallagher D Buckley M Kenny C , et al. A health screening and promotion clinic to improve metabolic monitoring for patients prescribed antipsychotic medication. Ir J Psychol Med. 2013;30(2):113–118. doi:10.1017/ipm.2013.5. 30199967

[28] Abdallah N Conn R Latif Marini A . Improving physical health monitoring for patients with chronic mental health problems who receive antipsychotic medications. BMJ Qual Improv Report. 2016;5(1):u210300.w4189. Epub ahead of print July 29, 2016. doi:10.1136/bmjquality.u210300.w4189. PMC4994095 27559474

[29] Soda T Richards J Gaynes BN , et al. Systematic quality improvement and metabolic monitoring for individuals taking antipsychotic drugs. PS (Wash DC). 2021;72(6):647–653. doi:10.1176/appi.ps.202000155. PMC8192348 33887956

[30] Gill M McKenna K McCauley M Gulzar M . Establishing a physical health monitoring service for patients on depot antipsychotic medication. Ir J Psychol Med. 2021;38(1):16–22. doi:10.1017/ipm.2016.41. 33715643

[31] Keenan R Chepulis L Ly J , et al. Metabolic screening in primary care for patients with schizophrenia or schizoaffective disorder and taking antipsychotic medication. J Prim Health Care. 2020;12(1):29–34. doi:10.1071/HC19023. 32223847

[32] Fischer SH Tjia J Reed G Peterson D Gurwitz JH Field TS . Factors associated with ordering laboratory monitoring of high-risk medications. J Gen Intern Med. 2014;29(12):1589–1598. doi:10.1007/s11606-014-2907-9. 24965280 PMC4242891

[33] Horn M Procyshyn RM Warburton WP , et al. Prescribing second-generation antipsychotic medications: practice guidelines for general practitioners. B C Med J. 2012;54(2):75.

[34] Stephenson E Yusuf A Gronsbell J , et al. Disruptions in primary care among people with schizophrenia in Ontario, Canada, during the COVID-19 pandemic. Can J Psychiatry. 2023;68(6):426–435. doi:10.1177/07067437221140384. 36453004 PMC9720063

[35] Panagiotopoulos C Ronsley R Davidson J . Increased prevalence of obesity and glucose intolerance in youth treated with second generation antipsychotic medications. Can J Psychiatry. 2009;54(11):743–749. doi:10.1177/070674370905401104. 19961662

[36] Sierra-Heredia C Tayyar E Bozorgi Y , et al. Growing inequities by immigration group among older adults: population-based analysis of access to primary care and return to in-person visits during the COVID-19 pandemic in British Columbia, Canada. BMC Prim Care. 2024;25(1):332. doi:10.1186/s12875-024-02530-1. 39243016 PMC11378608

[37] Charlson ME Pompei P Ales KL MacKenzie CR . A new method of classifying prognostic comorbidity in longitudinal studies: development and validation. J Chronic Dis. 1987;40(5):373–383. doi:10.1016/0021-9681(87)90171-8. 3558716

[38] Kurdyak P Lin E Green D Vigod S . Validation of a population-based algorithm to detect chronic psychotic illness. Can J Psychiatry. 2015;60(8):362–368. doi:10.1177/070674371506000805. 26454558 PMC4542516

[39] Bice TW Boxerman SB . A quantitative measure of continuity of care. Med Care. 1977;15(4):347–349. doi:10.1097/00005650-197704000-00010. 859364

[40] World Health Organization. Guidelines for ATC classification and DDD assignment 2023. Oslo, Norway: WHO Collaboration Centre for Drug Statistics Methodology; 2023.

[41] Ally BAS Stallman HM . Evaluation of a clozapine decision support tool in a mental health facility. J Pharm Pract Res. 2016;46(2):137–138. doi:10.1002/jppr.1208.

[42] Ng F Mammen OK Wilting I , et al. The International Society for Bipolar Disorders (ISBD) consensus guidelines for the safety monitoring of bipolar disorder treatments. Bipolar Disord. 2009;11(6):559–595. doi:10.1111/j.1399-5618.2009.00737.x. 19689501

[43] National Institute for Health and Care Excellence. Recommendations. Psychosis and schizophrenia in children and young people: recognition and management. Guidance. NICE. https://www.nice.org.uk/guidance/cg155/chapter/Recommendations (2013, accessed November 15, 2024).

[44] Chen W Cepoiu-Martin M Stang A , et al. Antipsychotic prescribing and safety monitoring practices in children and youth: a population-based study in Alberta, Canada. Clin Drug Investig. 2018;38(5):449–455. doi:10.1007/s40261-018-0626-4. 29453686

[45] Yang D Dalton JE A unified approach to measuring the effect size between two groups using SAS. SAS Glob Forum. 2012;335:1–6.

[46] SAS Institute Inc. 9.4 user’s guide. Cary, NC, USA; 2016.

[47] Kioko E Williams K Newhouse B . Improving metabolic syndrome screening on patients on second generation antipsychotic medication. Arch Psychiatr Nurs. 2016;30(6):671–677. doi:10.1016/j.apnu.2016.03.004. 27888958

[48] Feeney L Mooney M . Atypical antipsychotic monitoring in the Kilkenny Mental Health Services. Ir J Psychol Med. 2005;22(3):101–102. doi:10.1017/S0790966700009113. 30308759

[49] Dhamane AD Martin BC Brixner DI Hudson TJ Said Q . Metabolic monitoring of patients prescribed second-generation antipsychotics. J Psychiatr Pract. 2013;19(5):360–374. doi:10.1097/01.pra.0000435035.45308.03. 24042242

[50] Mahmood B Li G Li J , et al. Impact of the COVID-19 pandemic and control measures on screening and diagnoses of type 2 diabetes in British Columbia. Int J Environ Res Public Health. 2025;22(4):519. doi:10.3390/ijerph22040519. 40283745 PMC12026491

[51] Hafid S Freeman K Aubrey-Bassler K , et al. Describing primary care patterns before and during the COVID-19 pandemic across Canada: a quasi-experimental pre-post design cohort study using national practice-based research network data. BMJ Open. 2024;14(5):e084608. doi:10.1136/bmjopen-2024-084608. PMC11110591 38772895

[52] Ruangsomboon O Zhong A Kopp A , et al. Changes in primary care health services during the COVID-19 pandemic: a longitudinal analysis of data from Ontario. Healthcare Policy. 2024;19(4):42. 10.12927/hcpol.2024.27362 PMC11411643 39229662

[53] Stamenova V Chu C Pang A , et al. Virtual care use during the COVID-19 pandemic and its impact on healthcare utilization in patients with chronic disease: a population-based repeated cross-sectional study. PLoS ONE. 2022;17(4):e0267218. doi:10.1371/journal.pone.0267218. PMC9037937 35468168

[54] Maximova K Marashi M Holmes E , et al. Changes in chronic disease prevention resources and activities in Canada during the COVID-19 pandemic. Health Promot Chronic Dis Prev Can. 2025;45(7-8):335–344. doi:10.24095/hpcdp.45.7/8.03. 40833304 PMC12455906

[55] Shah TI Clark AF Seabrook JA Sibbald S Gilliland JA . Geographic accessibility to primary care providers: comparing rural and urban areas in Southwestern Ontario. Can Geogr / Géogr Can. 2020;64(1):65–78. doi:10.1111/cag.12557.

[56] Madore S Peterson S Rudoler D Lavergne R . Chronic disease management among people with serious mental illness across rural, small urban, and metropolitan settings. Ann Fam Med. 2024;22(Supplement 1):6943. Epub ahead of print November 20, 2024. doi:10.1370/afm.22.s1.6943.

[57] Webster P . Canada’s family physician shortage. Lancet. 2024;403(10441):2278. doi:10.1016/S0140-6736(24)01036-5. 38768629

[58] Rodrigues R Reid JNS Wiener JC , et al. Access to a regular primary care physician among young people with early psychosis in Ontario, Canada. Early Interv Psychiatry. 2024;18(7):513–523. doi:10.1111/eip.13487. 38036458

[59] Ali RA Jalal Z Paudyal V . Barriers to monitoring and management of cardiovascular and metabolic health of patients prescribed antipsychotic drugs: a systematic review. BMC Psychiatry. 2020;20(1):581. doi:10.1186/s12888-020-02990-6. 33276762 PMC7718699

[60] Poojari PG Khan S Shenoy S , et al. Perspectives of patients and healthcare professionals on metabolic monitoring of adult prescribed second-generation antipsychotics for severe mental illness: a meta-synthesis. PLoS ONE. 2023;18(4):e0283317. doi:10.1371/journal.pone.0283317. PMC10115273 37075039

[61] Mclenon J Rogers MAM . The fear of needles: a systematic review and meta-analysis. J Adv Nurs. 2019;75(1):30–42. doi:10.1111/jan.13818. 30109720

[62] Aouira N Khan S Heussler H Haywood A Karaksha A Bor W . Understanding the perspective of youths on undergoing metabolic monitoring while on second-generation antipsychotics: challenges, insight, and implications. J Child Adolesc Psychopharmacol. 2023;33(7):279–286. doi:10.1089/cap.2023.0016. 37504897

[63] Shin S Moon S Wang J Choi YJ . Impact of institutional quality improvement initiatives on metabolic monitoring in mental disorder in patients treated with antipsychotics: a meta-analysis of intervention studies. J Glob Health. 2024;14:04074. doi:10.7189/jogh.14.04074. 38783701 PMC11116930

[64] Ronsley R Rayter M Smith D Davidson J Panagiotopoulos C . Metabolic monitoring training program implementation in the community setting was associated with improved monitoring in second-generation antipsychotic-treated children. Can J Psychiatry. 2012;57(5):292–299. doi:10.1177/070674371205700504 22546061

[65] Immigration, Refugees and Citizenship Canada. Permanent resident database. Population Data BC; 2023. http://www.popdata.bc.ca/data

[66] BC Mental Health & Substance Use Services. Provincial Health Services Authority (PHSA) -BCMHSUS Tertiary Mental Health. 2022. DOI: Population Data BC. BCMHSUS. http://www.popdata.bc.ca/data

