# Supplementary material for: Monitoring Rates for Metabolic Syndrome in Adults Treated With Atypical Antipsychotics: A Population-Based Study in British Columbia: Fréquence de la surveillance du syndrome métabolique chez les adultes traités par des antipsychotiques atypiques : étude fondée sur la population menée en Colombie-Britannique
Source: Can J Psychiatry. 2026 Feb 13:07067437261420884. Online ahead of print. doi: 10.1177/07067437261420884 (PMC12904800; doi:10.1177/07067437261420884)
Supplement: sj-docx-1-cpa-10.1177_07067437261420884 - Supplemental material for Monitoring Rates for Metabolic Syndrome in Adults Treated With Atypical Antipsychotics: A Population-Based Study in British Columbia: Fréquence de la surveillance du syndrome métabolique chez les adultes traités par des antipsychoti [file sj-docx-1-cpa-10.1177_07067437261420884.docx]

List of ICD-9 and ICD-10 CA codes used for disorder groups - Supplemental material for Monitoring Rates for Metabolic Syndrome in Adults Treated with Atypical Antipsychotics: A Population-Based Study in British Columbia; Description

 Table S1 – List of ICD-9 and ICD-10 CA codes used for disorder groups

| Disorder group | Description | Data source | ICD-9 codes | ICD-10 CA |
| --- | --- | --- | --- | --- |
| Treated for schizophrenia, schzoaffective disorders, and psychotic disorder not otherwise specified | Flag individuals hospitalized (for any duration) with schizophrenia spectrum disorders as the most responsible diagnosis in DAD and/or at least three outpatient visits in MSP within a 36-month look-back period | Medical Services Plan (MSP)  Discharge Abstract Database (DAD)  British Columbia Mental Health and Substance Use Services (BCMHSUS) | 295 and 298 | F20, F25, F29 |
| Other conditions with psychosis | Flag individuals with one or more claims for a psychosis diagnosis in outpatient visits or diagnosis in DAD/BCMHSUS (any position) in a 36-month look-back period. | Medical Services Plan (MSP)  Discharge Abstract Database (DAD)  British Columbia Mental Health and Substance Use Services (BCMHSUS) | 290-294.9, 295, 298, 296, 297, 293.81, 293.82, 290-294.9 | F20, F25, F29, F21, F22, F23, F24, F28, F19.5, F30, F31x, F34 |
| Other mental illness or health conditions | Flag individuals who received any diagnosis other than bipolar disorders, schizophrenia spectrum and other psychotic diorders | Medical Services Plan (MSP)  Discharge Abstract Database (DAD)  British Columbia Mental Health and Substance Use Services (BCMHSUS) | 290-319, 327.x, 347.0, 347.1, 780.5x, V69.4  Exclude; 290-294.9, 295, 296, 297, 298, 293.81, 293.82 | F01-F99, G25.8, G47, G47.0, G47.1, G47.10-G47.14, G47.19, G47.2, G47.3, G47.30, G47.31, G47.310, G47.33, G47.34, G47.37, G47.38, G47.39, G47.4, G47.411, G47.419, G47.421, G47.429, G47.8, G47.9, R06.81  Exclude: F19.5, F20, F21, F22, F23, F24, F25, F28, F29, F30–F31, F34.0 |
